# Marker Selection in Multivariate Genomic Prediction Improves Accuracy of Low Heritability Traits

**DOI:** 10.3389/fgene.2020.499094

**Published:** 2020-10-30

**Authors:** Jaroslav Klápště, Heidi S. Dungey, Emily J. Telfer, Mari Suontama, Natalie J. Graham, Yongjun Li, Russell McKinley

**Affiliations:** ^1^Scion (New Zealand Forest Research Institute Ltd.), Rotorua, New Zealand; ^2^Skogforsk, Umeå, Sweden; ^3^Agriculture Victoria, AgriBio Center, Bundoora, VIC, Australia

**Keywords:** multivariate mixed model, genomic prediction, variable selection, PLS, *Pinus radiata*, *Eucalyptus nitens*

## Abstract

Multivariate analysis using mixed models allows for the exploration of genetic correlations between traits. Additionally, the transition to a genomic based approach is simplified by substituting classic pedigrees with a marker-based relationship matrix. It also enables the investigation of correlated responses to selection, trait integration and modularity in different kinds of populations. This study investigated a strategy for the construction of a marker-based relationship matrix that prioritized markers using Partial Least Squares. The efficiency of this strategy was found to depend on the correlation structure between investigated traits. In terms of accuracy, we found no benefit of this strategy compared with the all-marker-based multivariate model for the primary trait of diameter at breast height (DBH) in a radiata pine (*Pinus radiata*) population, possibly due to the presence of strong and well-estimated correlation with other highly heritable traits. Conversely, we did see benefit in a shining gum (*Eucalyptus nitens*) population, where the primary trait had low or only moderate genetic correlation with other low/moderately heritable traits. Marker selection in multivariate analysis can therefore be an efficient strategy to improve prediction accuracy for low heritability traits due to improved precision in poorly estimated low/moderate genetic correlations. Additionally, our study identified the genetic diversity as a factor contributing to the efficiency of marker selection in multivariate approaches due to higher precision of genetic correlation estimates.

## 1. Introduction

Heritability is one of the most important genetic parameters to consider for breeding, defined as the proportion of phenotypic variance explained by underlying genetic factors (Falconer and Mackay, 1996). Trait heritability is affected by changes in allelic frequencies due to selection or inbreeding, introduction of new alleles through mutation or migration (Latta, 2010), or due to changes in genetic effect due to altered genetic backgrounds or environmental conditions (Chandler et al., 2017). Quantitative traits normally present low to moderate heritability, as a result of their genetic control and the high degree of environmental influence on the expression of these traits. In tree breeding, important quantitative traits, such as height, diameter at breast height and stem volume generally have relatively low to moderate heritability estimates, ranging from 0.09 to 0.3 (Ukrainetz et al., 2008; Chen et al., 2018; Hayatgheibi et al., 2019). Furthermore, the magnitude and precision of these heritability estimates vary with the testing effort (such as sample size, experimental, and mating design) and the ontogenetic stage of individuals in the population being tested (Bouvet et al., 2003; Mihai and Mirancea, 2016). Reports of low heritability for productivity traits is not surprising, as they are assumed to be essential for individual tree survival and thus likely close to fixation (King, 1990; Merilä and Sheldon, 2000; Blows and Hoffmann, 2005). Unfortunately, both low heritability and less accurate estimates of breeding values makes selection decisions challenging for such traits and slows progress in genetic improvement.

The current rapid development of genomic resources in forest tree species (Neale and Kremer, 2011; Nystedt et al., 2013; Myburg et al., 2014; Neale et al., 2014) has improved forest tree breeding practices through the implementation of genomic prediction (Meuwissen et al., 2001; Grattapaglia and Resende, 2011; Isik, 2014; Grattapaglia et al., 2018). Genomic best linear unbiased prediction (GBLUP) is the most popular method for genomic prediction, due to the simple substitution of the average numerator relationship matrix (Wright, 1922) with a marker-based relationship matrix (Nejati-Javaremi et al., 1997; VanRaden, 2008; Hayes et al., 2009). Such a relationship matrix allows tracking of both recent and historical relatedness (Powell et al., 2010), as well as Mendelian segregation (Visscher et al., 2006) and linkage disequilibrium (LD) between markers and quantitative trait loci (QTLs) (Habier et al., 2013). The ultimate goal of genomic prediction is the development of model using mainly LD between markers and QTLs which would support predictive ability stable across generations. Sun et al. (2016) found that the accuracy of such model across generations is high only when the historical LD between markers and QTLs is high. Alternatively, the capture of co-segregation improves accuracy of the prediction when effective population is relatively small. Additionally, the accuracy of genomic prediction critically depends on the level of relatedness between the training and validation populations (Scutari et al., 2016).

While genetic correlations often represent evolutionary constraints (Clark, 1987), they are also a means to improve the accuracy of genetic parameters (Calus and Veerkamp, 2011) and reduce bias of estimated breeding values caused by selection on correlated trait through use of a multivariate instead of univariate approach (Pollak et al., 1984). The use of multivariate linear mixed models in genetic evaluations provides a basis for inference about traits' integration (Armbruster et al., 2014) as well as evolutionary response to selection (Sedlacek et al., 2016). Additionally, these types of models could deliver improvements in the accuracy of genetic parameters, especially where traits with low heritability can be analyzed together with traits of high heritability, and genetic covariances can be taken into consideration (Jia and Jannink, 2012; Marchal et al., 2016). Guo et al. (2014) reported an advantage to using multi-trait genomic predictions over single-trait alternatives when traits had low heritability or if phenotypic records were lacking. The traits with low heritability (Stejskal et al., 2018) benefited the most from the implementation of genomic information in the genetic analysis (Meuwissen et al., 2001). Therefore, a combination of both approaches in a genomic-based multivariate mixed linear model might provide the best results. However, both approaches have their drawbacks. Multivariate analysis can provide benefits to low heritability traits only in cases where there are strong genetic correlations with other traits, while no benefit or even reductions in breeding values accuracy can result when genetic correlations are weak (Jia and Jannink, 2012). Furthermore, optimization of the population sample size, effective population size and the level of genetic diversity captured is required to reach statistically significant genetic correlations (Bijma and Bastiaansen, 2014).

The majority of complex quantitative traits follows Fisher's infinitesimal model (Fisher, 1918) where each QTL contributes by only small fraction of total genetic variance. Such traits require genomic prediction models using large amount of genetic marker densely populating whole genome (Meuwissen et al., 2001; Guo et al., 2010). However, some traits show a positive response in prediction accuracy as a result of marker selection (Resende et al., 2012), depending on the structure of the training population and the genetic complexity of the investigated trait (Berger et al., 2015). Bayesian models have proven an efficient way to consider different variances for the distribution of marker effects which might result in an improvement in genomic predictions over classical GBLUP, especially in cases where the underlying genetic architecture of a trait involves large-effect QTLs (Cole et al., 2009).

Alternatively, construction of a trait-specific relationship matrix, considering marker-specific weights, provides a viable alternative (Zhang et al., 2010; Su et al., 2014). Lippert et al. (2013) investigated the ratio of causal and non-causal variants present in genomic data, and found that the most precise genetic parameter estimates are obtained when only causal variants are included in the prediction model. de los Campos et al. (2015) argued that a large number of markers in imperfect LD with QTLs can produce false inferences about heritability due to instability in likelihood estimates, especially when LD decays rapidly. Additionally, using an exhaustive amount of genomic information in genetic analyses can potentially reduce the precision of genetic parameters and the accuracy of genomic estimated breeding values (Habier et al., 2007, 2013).

Similar to single-trait genomic prediction models, several marker selection strategies have been developed within multi-trait genomic prediction models. Classical multiple regression models assign effects to every marker, which is not necessarily biologically true. Cheng et al. (2018), therefore, developed a Bayesian multi-trait model which allows for the assumption that each marker affects only one or a few traits, and has no effect on other traits. Karaman et al. (2018) applied an alternative approach using posterior estimates of marker effect covariances to weight their contribution to the marker-based relationship matrix, implemented in the GBLUP model. They found a further advantage to this weighted marker-based relationship matrix when weights were assigned to blocks of 100 SNPs, rather than to each marker separately.

The aim of this study is the improvement of genomic prediction for traits with relatively low heritability and poor prediction accuracy, such as those related to forest tree productivity (Gamal El-Dien et al., 2015; Ratcliffe et al., 2015), through the implementation of multi-trait models using a relationship matrix based only on prioritized markers. Our primary trait under investigation was diameter at breast height (DBH) for radiata pine (*Pinus radiata* D.Don) and shining gum [*Eucalyptus nitens* (H. Deane & Maiden) Maiden], a proxy for productivity in forest trees and thus considered the most economically important trait for those species. Non-target traits involved in the multivariate analysis represent operationally measured attributes related to stem form and wood quality.

## 2. Materials and Methods

### 2.1. Plant Material

#### 2.1.1. Radiata Pine (*Pinus radiata*)

The *P. radiata* population used in this study included 523 vegetatively propagated individuals (four ramets per individual genotype), structured into 42 full-sib families each represented by ~10 individual genotypes, part of The New Zealand Radiata Pine Breeding Company's (RPBC) program, selected for growth and form attributes. The field experiment was established as an incomplete block design containing nine blocks, each comprising six families with five replicates per family. All individuals were evaluated for the following traits: branch cluster frequency (BR9), visually assessed using a 9-point scale from 1 (uninodal) to 9 (extremely multinodal) (Carson, 1991); stem straightness (ST9), visually assessed using a 9-point scale from 1 (crooked) to 9 (very straight) (Carson, 1986); diameter at breast height (DBH [cm]) measured with diameter tape; wood density (WD, [kg/m^3^]), measured as basic wood density through the maximum moisture content method (Smith, 1954); and predicted modulus of elasticity (PME [GPa]), inferred from acoustic wave velocity using HITMAN (HM200) (Carter et al., 2007).

Genomic data were generated through an exome capture-based Genotype-By-Sequencing (GBS) platform (Neves et al., 2013), developed using in-house genomic resources (Telfer et al., 2018). The captured markers were filtered using a previously reported bioinformatics pipeline (Telfer et al., 2019). In brief, markers were removed if heterozygosity in haploid megagametophyte tissues was higher than 5%, average read depth was <10 (mean average read depth per marker was ~60 in our data) and have more than 1 alternative allele. Individual datapoints were classified as missing if the ratio between the reference and alternative allele was lower than 0.1 and the number of read was <10 (Telfer et al., 2019). In total, 80,160 SNPs passed the criteria, and were further filtered to remove SNPs with minor allele frequencies (MAF) <0.05 and a SNP call rate <0.6. The average proportion of SNP missing data was 9.9%. The genotype mean was used to impute missing data and 58,636 SNPs were used in downstream analysis.

#### 2.1.2. Shining Gum (*Eucalyptus nitens*)

The *E. nitens* population used in this study included 691 individuals, part of the third generation of open-pollinated progeny established within New Zealand's breeding program. The experimental design contained 30 replications of randomized complete blocks of these “sets” with each replication of the “set” comprising the same families but different individuals within these families (Klápště et al., 2019). Missing relatedness information in this population was recovered using sib-ship reconstruction as genomic information was not available for all possible parents (Klápště et al., 2017). This sib-ship reconstruction-based relationship matrix was used in both the genomic-based and pedigree-based scenarios in this study. The individuals within the open-pollinated progeny trial were phenotyped for diameter at breast height at age 6 (DBH [mm]) and for wood quality traits, such as wood density (WD [kg/m^3^]), wood stiffness (ST [km/s]), growth strain (GS [mm]), and average tangential air-dry shrinkage (TS [%]) measured on two different logs: log 1 from 1.4 to 3 m (index 1) and log 2 from 3 to 6 m (index 2) at the age of 7 (Klápště et al., 2017). Diameter at breast height was measured with diameter tape, wood density was measured as basic wood density through the maximum moisture content method (Smith, 1954), wood stiffness was measured indirectly as acoustic wave velocity using HITMAN (HM200) (Carter et al., 2007), growth strain was assessed by ripping logs with a chainsaw and measuring the resulting openings at the end of the log and average tangential air-dry shrinkage was measured following standard wood quality assessment protocols (Treloar and Lausberg, 1997).

Genomic data were generated using the EUChip60K SNP chip (Silva-Junior et al., 2015). SNP genotypes were called using the *Maidenaria* section specific cluster files (Silva-Junior et al., 2015) and filtered using Illumina metrics genTrain score >0.5 and GenCall >0.15, in addition to MAF >0.01 and call rate >0.6. The average proportion of SNP missing data was 5.8%. The genotype mean was used to impute missing data, with 9,697 SNPs used in downstream analysis.

### 2.2. Statistical Analysis

A univariate model was used to estimate variance components and derive narrow-sense heritability for both species using the following mixed linear model implemented in statistical package ASReml-R (Butler et al., 2009):

y=Xβ+Zg+Zb+e

where ***y*** is the vector of individual-tree trait measurements, ***β*** is the vector of fixed effects (intercept and replicate, as well as seed orchard in the case of *E.nitens*), ***g*** is the vector of random additive genetic values following var(***g***)~N(0, ***A***$\sigma_{g}^{2}$), where $\sigma_{g}^{2}$ is the genotypic variance and ***A*** is the average numerator relationship matrix (Wright, 1922), ***b*** is the vector of random block effects nested within replication effects following var(***b***)~N(0, ***I***$\sigma_{b}^{2}$), where $\sigma_{b}^{2}$ is block nested within replication variance, ***e*** is the vector of random residual effects following var(***e***)~N(0, ***I***$\sigma_{e}^{2}$), and where $\sigma_{e}^{2}$ is the residual variance.

Additionally, a univariate model was used to estimate best linear unbiased estimates (BLUEs) for genotype in *P. radiata* as well as to correct phenotypes for design effects in the *E. nitens* population using the following mixed linear model implemented in statistical package ASReml-R (Butler et al., 2009):

y=Xβ+e

where ***y*** is the vector of individual-tree trait measurements, ***β*** is the vector of fixed effects (intercept, replicates and block nested within replicates, and genotype in the case of *P. radiata*), ***e*** is the vector of random residual effects following var(***e***)~N(0, ***I***$\sigma_{e}^{2}$), and where $\sigma_{e}^{2}$ is the residual variance.

The BLUE estimates for genotypes for *P. radiata* and corrected phenotypes for *E. nitens* were used along with the genomic data to estimate marker weights prior to construction of the marker-based relationship matrix. Weights for marker selection were derived through two blocks of canonical partial least squares (PLS-CA) (Tenenhaus, 1998) implemented using the “plsca” function from the R package “plsdepot” (Sanchez and Sanchez, 2012). The algorithm computes sequences of pairs of vectors of latent scores which are orthogonal by maximization of Cov(***Xu***, ***Yv***), where ***X*** is the scaled matrix of marker genotypes and ***Y*** is the scaled matrix of clonal values for measured traits, and ***u*** and ***v*** are vectors of coefficients maximizing the covariance. The coefficients in ***u*** measure the importance of variables in ***X*** (genetic markers) to latent variables, and were therefore used as criteria for selection of markers to calculate the marker-based relationship matrix. Since prior knowledge of genetic architecture in studied traits and complexity of pleiotropy and QTL collocation is usually lacking, exploration of the whole matrix of combinations of selection intensity for potentially informative genetic markers was required. First, marker coefficients in the vector ***u*** associated with each component were truncated by the 90th, 80th, 70th, 60th, and 50th percentiles, and loadings for selected markers were transformed to either 1 or 0. For each percentile level, different numbers of components were included into the marker selection process.

Univariate models using corrected phenotypes and pedigree (BLUP) or marker information (GBLUP) were used to estimate narrow-sense heritability (the proportion of additive to total genetic variance in the case of *P. radiata*) and prediction accuracy using the “BGLR” statistical R package (Pérez and de Los Campos, 2014), as follows:

y=Xβ+Zg+e

where *y* is the vector of corrected phenotypes/genotypic values, ***β*** is the vector of fixed effects (overall mean), ***g*** is the vector of additive genetic effects following var(g)~N(0, ***A***$\sigma_{g}^{2}$), where ***A*** is the average numerator relationship matrix (Wright, 1922) in the BLUP analysis, and is substituted by marker-based relationship ***G*** (VanRaden, 2008) in the GBLUP analysis, $\sigma_{g}^{2}$ is additive genetic variance, ***e*** is the vector of residuals following var(e)~N(0, ***I***$\sigma_{e}^{2}$), where ***I*** is the identity matrix and $\sigma_{e}^{2}$ is residual variance.

Since the aim of the algorithm is the maximization of covariance among genomic and phenotypic data, the first scenario selects only markers with the highest positive coefficients, which have an associated positive effect with the underlying covariance/correlation structure (positive pleiotropy) (scenario MVGBLUP1). However, the relationship between traits is not driven only by markers acting in the same direction; some markers act in the same direction only for certain sets of traits, and in opposite directions for other traits (negative pleiotropy). To investigate the impact of such markers, we tested a second scenario where markers involved in the construction of the relationship matrix were selected from both positive and negative tails of the loading distribution (scenario MVGBLUP2). For example, in the 90th percentile scenario, markers were selected from both above the 90th percentile and from below the 10th percentile. The other scenarios continued to select the markers having loadings closer to the middle of their distribution. Again, this marker selection strategy was applied across the variable number of components included in this study. The improved marker-based estimates of genetic correlation were performed using marker weights implemented in the construction of a trait-specific marker-based relationship matrix (Zhang et al., 2010) as follows:

$$G_{w}=\frac{ZWZ^{\prime }}{\sum w_{i}}$$

where ***G***_****w****_ is the marker-based relationship matrix, ***Z*** = ***M*** − ***P***, where ***M*** is the matrix of genotypes coded as 0, 1, and 2 for reference allele homozygotes, heterozygotes and the alternative allele homozygotes, respectively, ***P*** is the vector of doubled allelic frequencies for the alternative allele, ***W*** is the diagonal matrix of weights and *w*_*i*_ is the weight for the ith marker. The effect of SNP selection on the precision of genetic parameters and prediction accuracy of genomic estimated breeding values was investigated through multivariate mixed linear modeling using Gibbs sampling, performed in the “MTM” package (de los Campos and Grüneberg, 2016) implementing algorithms from the “BGLR” statistical R package (Pérez and de Los Campos, 2014), as follows:

Y=Xβ+Za+e

where ***Y*** is a matrix of phenotypes, ***a*** is the vector of random genomic breeding values following var(***a***)~N(0,G1), where G1 is a variance-covariance structure for additive genetic effects following G1=$\left[\begin{array}{ccc} \sigma_{a_{1}}^{2} & \ldots & \sigma_{{\text{a}}_{1}{\text{a}}_{\text{n}}}\\ \vdots & \ddots & \vdots \\ \sigma_{{\text{a}}_{\text{n}}{\text{a}}_{1}} & \ldots & \sigma_{a_{\text{n}}}^{2} \end{array}\right]$ ⊗ ***G***, where $\sigma_{a_{1}}^{2}$ and $\sigma_{a_{\text{n}}}^{2}$ are additive genetic variances for the 1st and nth trait, respectively, σ_a_1_a_n__ and σ_a_n_a_1__ are additive genetic covariances between the 1st and nth trait, ⊗ is the Kronecker product and ***G*** is the marker-based relationship matrix estimated either as follows:

$$G=\frac{ZZ^{\prime }}{2\sum p_{\text{i}}\left(1-p_{\text{i}}\right)}$$

where p_i is the frequency of the alternative allele at the ith loci, or estimated on the basis of weighted markers (***G***_***w***_) as defined above, ***e*** is the vector of random residual effects following var(***e***)~N(0,R), where R is the residual variance-covariance structure following R = $\left[\begin{array}{ccc} \sigma_{e_{1}}^{2} & \ldots & \sigma_{{\text{e}}_{1}{\text{e}}_{\text{n}}}\\ \vdots & \ddots & \vdots \\ \sigma_{{\text{e}}_{\text{n}}{\text{e}}_{1}} & \ldots & \sigma_{e_{\text{n}}}^{2} \end{array}\right]$ ⊗ ***I***, where $\sigma_{e_{1}}^{2}$ and $\sigma_{e_{\text{n}}}^{2}$ are residual variances for the 1st and nth trait, and σ_e_1_e_n__ and σ_e_n_e_1__ are residual covariances between the 1st and nth trait. The number of iterations in BGLR was set to 300,000, with a burn-in period of 50,000 iterations, thinning to 10. Given the different percentiles of marker loadings and numbers of latent variables used in marker selection, the best scenario was identified on the basis of the deviance information criterion (DIC). Additionally, single-trait model (scenarios BLUP and GBLUP) were implemented for each investigated trait to evaluate the benefit of the multivariate model over univariate analysis. Trait heritability was estimated following:

$$h^{2}=\frac{\sigma_{a}^{2}}{\sigma_{a}^{2}+\sigma_{e}^{2}}$$

where $\sigma_{a}^{2}$ is additive genetic and $\sigma_{e}^{2}$ is residual variance. Genetic correlations were estimated through Pearson's product moment as follows:

$$r_{\text{G}}=\frac{\sigma_{{\text{a}}_{\text{x}}{\text{a}}_{\text{y}}}}{\sqrt{\sigma_{a_{\text{x}}}^{2}\sigma_{a_{\text{y}}}^{2}}}$$

where σ_a_x_a_y__ is the additive genetic covariance between the *x*th and *y*th trait, and $\sigma_{a_{\text{x}}}^{2}$ and $\sigma_{a_{\text{y}}}^{2}$ are the additive genetic variances for the xth and yth trait, respectively. The multivariate scenarios using all available markers (MVGBLUP) or pedigree/sib-ship reconstruction (MVBLUP) were considered as benchmarks in this study.

Independent evaluation was performed using a 10-fold cross-validation. Nine-folds formed the training population, where PLS-CA was performed to obtain marker weights and construct the marker-based matrix from selected markers. The 10th-fold was used as the validation population to predict genomic breeding values (GEBV). The prediction accuracy was estimated as correlations between EBVs and GEBVs predicted through cross-validation. The statistical significance of difference in prediction accuracy between benchmark and the best scenario using selected markers, non-parametric Wilcoxon rank test was implemented (Wilcoxon, 1992).

## 3. Results

### 3.1. Genetic Parameters

Discriminant analysis of principal components (DAPC) (Jombart et al., 2010) was performed to investigate population structure. We found almost no support for population stratification in *E. nitens* and scenario with two clusters showed the best fit of the data (Supplementary Figure 1). This scenario identified clusters associated to the each seed orchard progeny. The same approach applied in *P. radiata* selected seven clusters as the best scenario considering fit of the data (Supplementary Figure 1). The exploration of marker-based relationship matrices within each population through principal component analysis (PCA) found relatively weak stratification, mostly due to the separation of families accounting for 1.5–2.04% (*E. nitens*) and 3.44–3.79% (*P. radiata*) of the total variance attributed to the first two principal components (Supplementary Figure 2, upper plots). The distribution of relatedness showed that the majority of matrix elements had no or very weak relatedness. Additionally, there is a peak around 0.2, representing half-sibs in the *E. nitens* population, and two peaks around 0.2 and 0.4 in the *P. radiata* population, representing half-sibs and full-sibs (Supplementary Figure 2, bottom plots) corresponding to the mating strategy implemented at each population. The mean sample observed heterozygosity was ~0.29 in *E. nitens* and ~0.19 in *P. radiata*. The self-relatedness was distributed around 1 in *P. radiata*, but shifted to around 0.75 in *E. nitens* due to the higher level of inbreeding (Supplementary Figure 3).

Trait heritabilities were estimated using variance components inferred from a sib-ship reconstruction-based (BLUP) as well as marker-based (GBLUP) univariate model in *E. nitens*, and from a pedigree-based (BLUP) as well as marker-based (GBLUP) univariate model in *P. radiata*. Heritability estimates were moderate to high, ranging from 0.093 (ST2) to 0.282 (WD) using sib-ship (BLUP) and from 0.089 (DBH) to 0.559 (WD) using markers (GBLUP) in *E. nitens*, and from 0.046 (ST9) to 0.588 (WD) using pedigree (BLUP) and from 0.126 (ST9) to 0.529 (WD) using markers (GBLUP) in *P. radiata* (Table 1). In general, marker-based analysis (GBLUP) resulted in higher heritability estimates than pedigree/sib-ship based (BLUP) analysis.

**Table 1:** Heritability estimates and their 95% confidence limits using variance components inferred from the sib-ship reconstruction-based univariate model (BLUP) in *E. nitens* and from using the pedigree-based univariate model (BLUP) in *P. radiata* as well as marker-based univariate models (GBLUP).

	***E. nitens***		***P. radiata***	
**Trait**	**Pedigree**	**Markers**	**Pedigree**	**Markers**
TS	0.242 (0.147–0.338)	0.539 (0.389–0.689)	NA	NA
WD	0.282 (0.193–0.371)	0.559 (0.420–0.699)	0.588 (0.292–0.884)	0.529 (0.400–0.658)
DBH	0.138 (0.030–0.245)	0.089 (−0.049–0.228)	0.134 (0.024–0.244)	0.131 (0.052–0.210)
ST1	0.210 (0.107–0.313)	0.394 (0.229–0.559)	NA	NA
ST2	0.093 (−0.001–0.187)	0.199 (0.044–0.354)	NA	NA
GS1	0.248 (0.139–0.357)	0.309 (0.149–0.469)	NA	NA
GS2	0.211 (0.103–0.319)	0.318 (0.154–0.481)	NA	NA
ST9	NA	NA	0.046 (−0.010–0.102)	0.126 (0.034–0.218)
BR9	NA	NA	0.128 (0.019–0.237)	0.177 (0.073–0.282)
PME	NA	NA	0.224 (0.055–0.393)	0.397 (0.250–0.544)

*NA represents the case where data were not available for a particular species and trait*.

**Figure 1:**
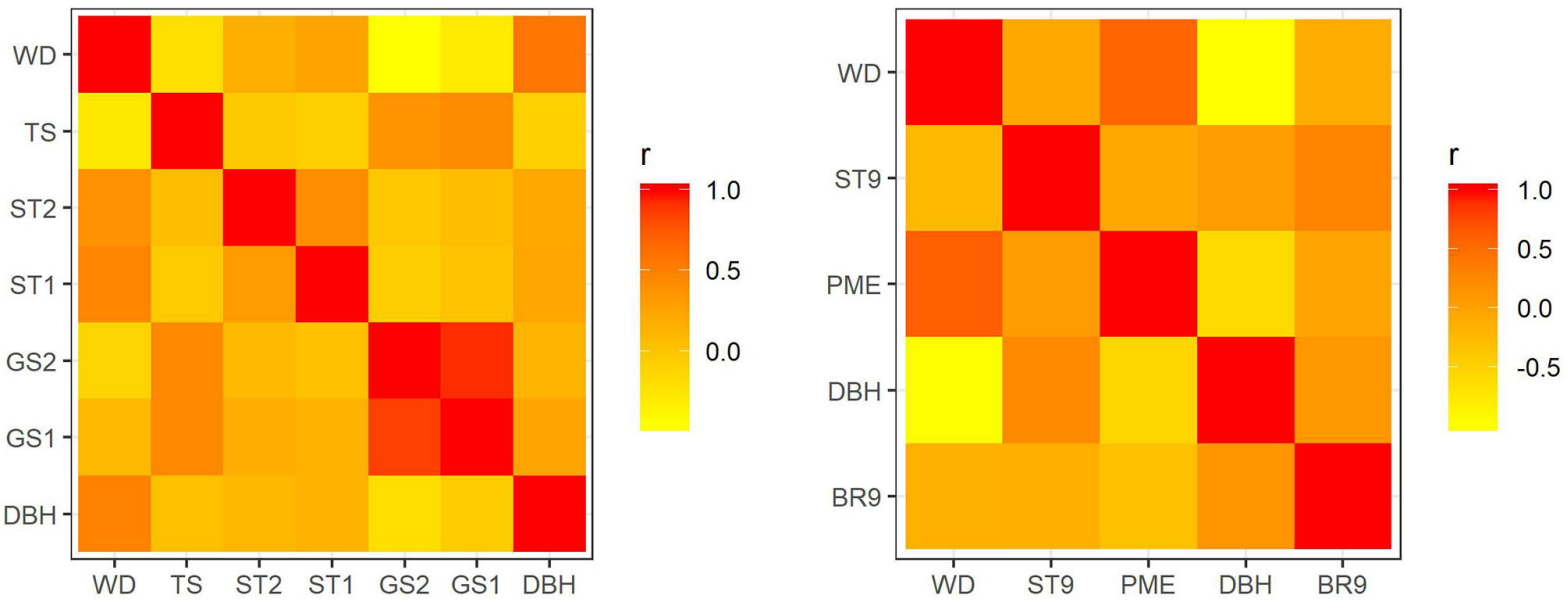
Genetic correlations using variance components and covariances inferred from use of a sib-ship reconstruction-based multivariate model (MVBLUP) (below diagonals) and marker-based relationship matrix (MVGBLUP) (above diagonals) in the *E. nitens* population (left plot) and using variance components and covariances inferred from use of a pedigree-based multivariate model (MVBLUP) (below diagonals) and marker-based relationship matrix (MVGBLUP) (above diagonals) in the *P. radiata* population (right plot).

**Figure 2:**
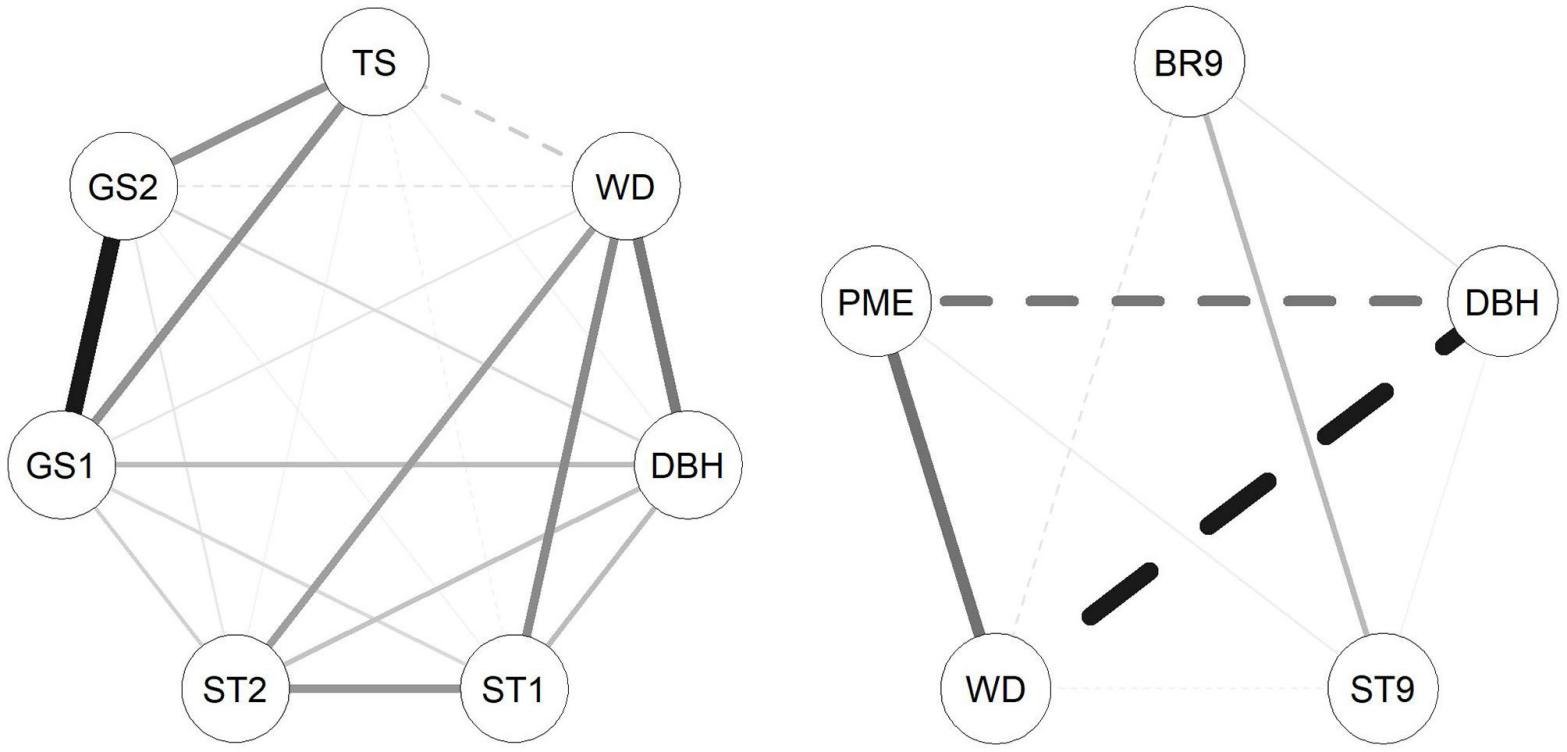
Correlation networks between traits investigated in the *E. nitens* (left) and *P. radiata* (right) populations based on genetic correlations estimated in multivariate model using marker-based relationship matrix. Solid lines represent positive correlations and dashed lines represent negative genetic correlations; the thickness of the lines represents magnitude of correlations.

In *E. nitens*, genetic correlations ranged from −0.459 (between WD and GS2) to 0.859 (between GS1 and GS2) using sib-ship (MVBLUP) (Figure 1—left plot below diagonals), and from −0.113 (between WD and GS2) to 0.929 (between GS1 and GS2) using markers (MVGBLUP) (Figure 1—left plot above diagonals). In *P. radiata*, genetic correlations ranged from −0.978 (between DBH and WD) to 0.548 (between WD and PME) using the pedigree (MVBLUP) (Figure 1—right plot below diagonals), and from −0.987 (between DBH and WD) to 0.602 (between WD and PME) using markers (MVGBLUP) (Figure 1—right plot above diagonals). Genetic correlations showed a more complex pattern in *E. nitens* compared with *P. radiata* (Figure 2).

### 3.2. Marker Selection

Using PLS-CA resulted in the construction of marker-based relationship matrices using different numbers of markers. When only markers with positive loadings (MVGBLUP1) were used, the number of selected markers ranged from 970 to 9,627 in *E. nitens* and from 5,864 to 56,809 in *P. radiata*. Scenarios which considered markers with both positive and negative loadings (MVGBLUP2) resulted in the number of selected markers ranging from 1,940 to 9,697 in *E. nitens* and from 9,838 to 58,636 in *P. radiata* (Table 2).

**Table 2:** Number of markers selected in different scenarios using only positive (upper part) or both positive and negative (bottom part) marker loadings obtained from PLS-CA procedure.

	**Species**	***E. nitens***					***P. radiata***				
**Scen**	**Prop**	**P10**	**P20**	**P30**	**P40**	**P50**	**P10**	**P20**	**P30**	**P40**	**P50**
	C1	970	1,940	2,909	3,879	4,849	5,864	11,728	17,591	23,455	29,318
	C2	1,824	3,513	4,999	6,292	7,348	11,364	21,448	30,668	38,650	45,014
	C3	2,574	4,704	6,371	7,634	8,510	15,856	28,529	38,725	46,448	51,793
Pos	C4	3,318	5,776	7,456	8,555	9,180	20,773	35,762	45,963	52,179	55,740
	C5	3,898	6,492	8,049	8,992	9,419	24,128	39,697	49,288	54,523	57,198
	C6	4,515	7,188	8,631	9,312	9,567	NA	NA	NA	NA	NA
	C7	4,997	7,632	8,896	9,452	9,627	NA	NA	NA	NA	NA
	C1	1,904	3,840	5,825	7,792	9,659	10,574	22,848	35,438	47,511	58,636
	C2	3,377	6,131	8,103	9,282	9,697	19,871	37,712	49,848	56,706	58,636
Pos	C3	4,578	7,502	9,048	9,612	9,697	2,8337	46,493	55,238	58,271	58,636
+	C4	5,558	8,314	9,418	9,680	9,697	34,662	51,277	57,188	58,542	58,636
Neg	C5	6,303	8,801	9,562	9,694	9,697	40,064	54,329	58,047	58,618	58,636
	C6	6,946	9,108	9,639	9,696	9,697	NA	NA	NA	NA	NA
	C7	7,425	9,300	9,665	9,697	9,697	NA	NA	NA	NA	NA

*NA represents the case not applicable for a particular species*.

The most intensive marker selection in the *E. nitens* population resulted in the worst model fit in terms of deviance information criteria (DIC). The model fit continually improved with more relaxed parameters on marker loadings. This pattern was observed for both tested strategies (MVGBLUP1 and MVGBLUP2). The best scenario appeared close to the one using all markers (MVGBLUP) (using seven components and the 40th percentile) (Supplementary Table 1). There was no real pattern to the number of markers selected in the *P. radiata* population, with the best model fit found for the scenario that used four latent components and the 50th percentile (Supplementary Table 1).

**Figure 3:**
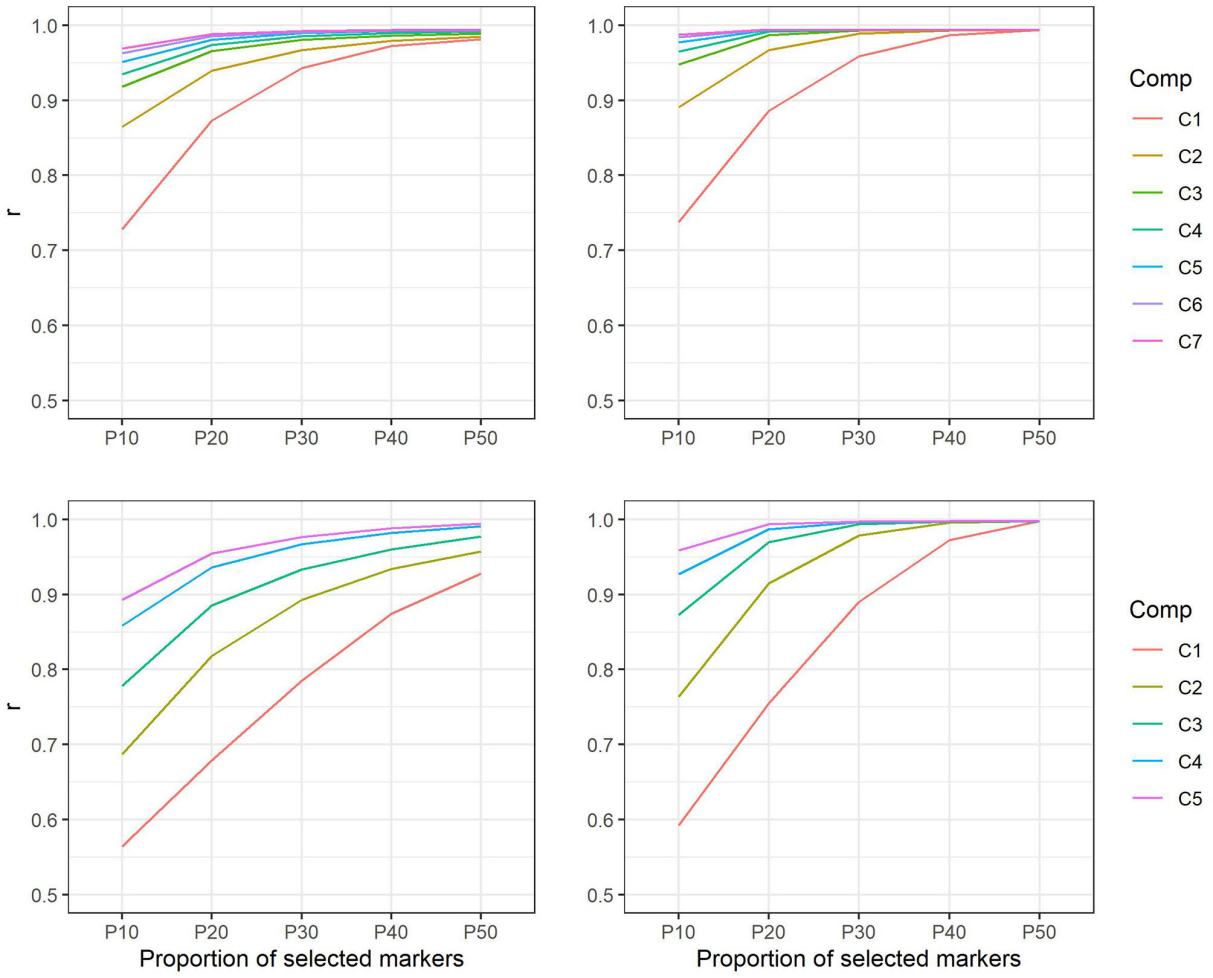
Correlations between marker-based relationship matrices using markers selected on the basis of positive loadings only and marker-based relationship matrix using all markers in *E. nitens* (upper left) and in *P. radiata* (bottom left) populations and correlations between marker-based relationship matrices using markers selected on the basis of both positive and negative loadings and marker-based relationship matrix using all markers in *E. nitens* (upper right) and in *P. radiata* (bottom right) populations. Each line represent scenario for different number of latent variables considered in marker selection (e.g., C1—only the first latent variable is considered, C2—only the first two latent variables are considered, etc.).

Comparison of the marker-based relationship matrix using all markers with matrices using only selected subsets of markers showed correlations from 0.73 to 0.99 in *E. nitens*. Similarly, in *P. radiata*, correlations reached values from 0.57 to 0.99. In both populations, the genetic correlations increased as the number of components as well as the proportion of markers selected within components increased (Figure 3).

### 3.3. Prediction Accuracy

Prediction accuracy in the pedigree/sib-ship based model (BLUP) ranged from 0.246 (DBH) to 0.782 (WD) in *E. nitens*, and from 0.441 (DBH) to 0.653 (BR9) in *P. radiata*. In marker-based models (GBLUP), this ranged from 0.183 (DBH) to 0.764 (WD) in *E. nitens*, and from 0.388 (DBH) to 0.645 (WD) in *P. radiata*. In general, the implementation of single-trait models (BLUP and GBLUP) resulted in lower prediction accuracies when the marker-based model (GBLUP) was compared to the pedigree/sib-ship based model (BLUP) (Tables 3, 4).

**Table 3:** Prediction accuracies and their standard deviations (in parenthesis) obtained from multivariate mixed models in the *E. nitens* population when using, a relationship matrix derived from sib-ship reconstruction (MVBLUP), a marker-based relationship matrix using all markers (MVGBLUP), a marker-based relationship matrix using selected SNPs having only positive loadings (MVGBLUP1), or a marker-based relationship matrix using selected SNPs having both positive and negative loadings (MVGBLUP2).

**Trait**	**BLUP**	**GBLUP**	**MVBLUP**	**MVGBLUP**	**MVGBLUP1**	**MVGBLUP2**
TS	0.737 (0.039)	0.656 (0.069)	0.754 (0.034)	0.665 (0.071)	0.650^NS^ (0.047)	0.642^NS^ (0.059)
WD	0.782 (0.060)	0.764 (0.054)	0.658 (0.068)	0.768 (0.049)	0.759^NS^ (0.053)	0.766^NS^ (0.035)
DBH	0.246 (0.132)	0.183 (0.117)	0.541 (0.251)	0.529 (0.336)	0.576^NS^ (0.241)	0.595^**^ (0.353)
ST1	0.613 (0.056)	0.523 (0.098)	0.621 (0.072)	0.545 (0.085)	0.525^NS^ (0.074)	0.523^NS^ (0.078)
ST2	0.571 (0.140)	0.448 (0.131)	0.582 (0.137)	0.442 (0.134)	0.434^NS^ (0.137)	0.414^NS^ (0.107)
GS1	0.683 (0.045)	0.558 (0.071)	0.720 (0.062)	0.609 (0.082)	0.604^NS^ (0.072)	0.604^NS^ (0.085)
GS2	0.603 (0.068)	0.547 (0.076)	0.737 (0.068)	0.651 (0.081)	0.650^NS^ (0.065)	0.660^NS^ (0.073)

*Predicted EBV/GEBVs were correlated with EBVs estimated when using the multivariate mixed model using either documented pedigree or relationships inferred from sib-ship reconstruction*.

** *represents a statistically significant while NS represents a statistically non-significant test at α level 0.05*.

**Table 4:** Prediction accuracies and their standard deviations (in parenthesis) obtained from multivariate mixed model in *P. radiata* population when using the documented pedigree (MVBLUP), a marker-based relationship matrix using all markers (MVGBLUP), a marker-based relationship matrix using selected SNPs having only positive loadings (MVGBLUP1), or a marker-based relationship matrix using selected SNPs having both positive and negative loadings (MVGBLUP2).

**Trait**	**BLUP**	**GBLUP**	**MVBLUP**	**MVGBLUP**	**MVGBLUP1**	**MVGBLUP2**
BR9	0.653 (0.088)	0.550 (0.121)	0.679 (0.095)	0.570 (0.136)	0.586^NS^ (0.134)	0.589^NS^ (0.123)
DBH	0.441 (0.103)	0.388 (0.133)	0.573 (0.069)	0.611 (0.062)	0.616^NS^ (0.058)	0.626^NS^ (0.048)
ST9	0.638 (0.147)	0.415 (0.148)	0.646 (0.126)	0.435 (0.135)	0.446^NS^ (0.149)	0.436^NS^ (0.119)
WD	0.642 (0.043)	0.645 (0.056)	0.610 (0.045)	0.618 (0.064)	0.627^NS^ (0.064)	0.631^NS^ (0.044)
PME	0.565 (0.118)	0.554 (0.119)	0.553 (0.109)	0.530 (0.116)	0.542^NS^ (0.113)	0.543^NS^ (0.108)

**represents a statistically significant while NS represents a statistically non-significant test at α level 0.05.

The prediction accuracies from the multi-trait model (MVBLUP and MVGLUP) were higher compared to the single-trait model (BLUP and GBLUP). Prediction accuracy in the pedigree/sib-ship based model (MVBLUP) ranged from 0.541 (DBH) to 0.754 (WD) in *E. nitens*, and from 0.553 (PME) to 0.679 (BR9) in *P. radiata*. In the marker-based model (MVGBLUP), this ranged from 0.529 (DBH) to 0.768 (WD) in *E. nitens*, and from 0.435 (ST9) to 0.618 (WD) in *P. radiata*. Generally, the implementation of multi-trait models (MVBLUP and MVGBLUP) followed a similar pattern as the single-trait model, in that the pedigree/sib-ship based model (MVBLUP) mostly outperformed the marker-based model (MVGBLUP), with a few exceptions, such as WD in *E. nitens* and DBH and WD in *P. radiata* (Tables 3, 4).

**Figure 4:**
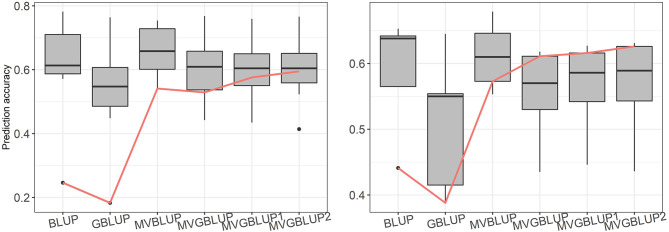
Boxplot of prediction accuracies for each tested scenario in *E. nitens* (left plot) and in *P. radiata* (right plot) populations, red line represents prediction accuracy for primary trait (DBH).

Prediction accuracy of the models with markers selected using only positive loadings (MVGBLUP1) ranged from 0.434 (ST2) to 0.759 (WD) in *E. nitens* and from 0.446 (ST9) to 0.627 (WD) in *P. radiata*. For models with markers selected using both positive and negative loadings (MVGBLUP2), prediction accuracies ranged from 0.414 (ST2) to 0.766 (WD) in *E. nitens*, and from 0.436 (ST9) to 0.631 (WD) in *P. radiata*. The marker-based models using marker selection (MVGBUP1 and MVGBLUP2) resulted in increased prediction accuracy of the primary trait while maintaining similar accuracies for other traits in *E. nitens*. No impact of marker-selection on prediction accuracy of the primary trait was observed in *P. radiata* (Tables 3, 4, Figure 4). The highest prediction accuracy for each trait was obtained using different marker selection scenarios, with no one scenario allowing for the highest prediction accuracy in all investigated traits simultaneously (Supplementary Tables 2–5). The significance of the improvement in prediction accuracy through marker selection was tested with the Wilcoxon non-parametric test, and a significant improvement was found only for DBH in *E. nitens* when the MVGBLUP2 model was implemented (Table 3).

The prediction accuracies estimated for each trait and marker selection scenario were correlated with DIC and number of selected markers. The correlations between prediction accuracy and DIC were strong for *E. nitens*, reaching values from −0.952 (TS) to −0.559 (DBH) in scenarios where marker selection was based on positive marker loadings, and from −0.951 (TS) to −0.332 (WD) in scenarios where marker selection was based on both positive and negative marker loadings. The correlations between prediction accuracy and DIC were relatively weak in *P. radiata* reaching values from −0.721 (WD) to 0.115 (BR9) in scenarios where marker selection was based on positive marker loadings, and from −0.583 (DBH) to 0.623 (BR9) in scenarios where marker selection was based on both positive and negative marker loadings.

The correlations between prediction accuracy and number of selected markers were strong in *E. nitens*, reaching values from 0.467 (DBH) to 0.910 (ST1) in scenarios where marker selection was based on positive marker loadings and from 0.274 (WD) to 0.923 (TS) in scenarios where marker selection was based on both positive and negative marker loadings. Conversely, the correlations between prediction accuracy and number of selected markers were rather weak in *P. radiata* reaching values from −0.235 (BR9) to 0.841 (DBH) where marker selection was based on positive marker loadings and from −0.613 (BR9) to 0.439 (DBH) where marker selection was based on both positive and negative marker loadings. For our primary trait (DBH), in both species the opposite pattern was found between prediction accuracy and number of selected markers compared with prediction accuracy and DIC (Table 5).

**Table 5:** Correlations between prediction accuracy and Deviance Information Criterion (DIC) and between prediction accuracy and number of selected markers.

	***E. nitens***				***P. radiata***			
**Trait**	**Pos**		**Pos + Neg**		**Pos**		**Pos + Neg**	
	**DIC**	**N_**Markers**_**	**DIC**	**N_**Markers**_**	**DIC**	**N_**Markers**_**	**DIC**	**N_**Markers**_**
TS	−0.952	0.849	−0.951	0.923	NA	NA	NA	NA
WD	−0.702	0.544	−0.332	0.274	−0.650	0.551	−0.557	0.364
DBH	−0.559	0.467	−0.409	0.358	−0.664	0.841	−0.583	0.439
ST1	−0.955	0.910	−0.902	0.855	NA	NA	NA	NA
ST2	−0.582	0.657	−0.455	0.504	NA	NA	NA	NA
GS1	−0.906	0.777	−0.756	0.701	NA	NA	NA	NA
GS2	−0.905	0.816	−0.635	0.600	NA	NA	NA	NA
ST9	NA	NA	NA	NA	−0.223	0.029	0.147	0.246
BR9	NA	NA	NA	NA	0.115	−0.235	0.623	−0.613
PME	NA	NA	NA	NA	−0.721	0.251	−0.194	0.082

*NA represents the case where no data were available for particular species and trait*.

## 4. Discussion

### 4.1. Effect of Phenotypic Integration

Any complex trait is the end-product of many pathways, with many of the genes involved contributing to multiple pathways (i.e., pleiotropy). The efficient coordination of the pathways responsible for each particular attribute requires a certain level of organization in space and time, developed through modularity in the biological processes (Wagner et al., 2007). Therefore, pathways to achieving certain phenotypic characteristics can be structured into different modules comprising a number of different levels of shared pathways. The characteristics within each module show a high level of phenotypic integration while the characteristics from different modules show a low level of integration (Wagner et al., 2007; Armbruster et al., 2014). Such stratification allows for effective independent evolution between modules, while the genetic correlations within the modules represent evolutionary constraints (Clark, 1987).

We proposed searching for markers that represent genomic regions involved in the shared pathways underlying the traits of interest. Our strategy for identifying such markers was through the alignment of the covariance structure within traits with the covariance structure within genetic markers, using a PLS-CA approach. This creates latent variables that collectively represent the studied attributes at each block (phenotypes on one side and genetic markers on the other side) through their shared variances (i.e., covariances). Since the method maximizes covariance between the latent variables from each block (phenotypes vs. genetic markers) through the coefficients in vectors ***u*** and ***v***, it is possible to emphasize the shared variance caused by genetics (i.e., the part of the phenotypic covariance associated with genetic markers). Markers with strong associations to this alignment (large loadings) are likely positioned within the genomic regions showing pleiotropy or an accumulation of QTLs responsible for studied traits. Due to evolutionary trade-offs of gene functions on overall fitness, pleiotropy can act in opposite directions for affected traits (Guillaume and Otto, 2012). As a result, markers with negative association with the alignment (large negative loadings) are also likely to be involved in the underlying genetic architecture of covariances between traits. Watanabe et al. (2019) found that 90% of genes identified in human genome-wide association studies (GWAS) were associated with multiple traits, emphasizing how commonly pleiotropy plays a part in the genetic architecture of complex traits. However, where the complexity of genetic covariances between studied traits is unknown, a range of selection intensity in genetic markers is needed. We thus adopted a marker selection strategy based on quantiles derived from the distribution of their loadings.

Our analysis found there was a benefit to using marker selection (MVGBLUP1 and MVGBLUP2) in the multivariate analysis in the *E. nitens* population. Including more traits with no strong relationships (Figure 1—left plot) increased the prediction accuracy for DBH beyond that observed for the model using all available markers. On the other hand, using a multivariate model with marker selection (MVGBLUP1 and MVGBLUP2) in the *P. radiata* population did not improve prediction accuracy of low heritability DBH beyond that observed for the model using all available markers, possibly due to strong genetic correlation between DBH and WD (Figure 1—right plot). Since the precision of genetic correlations estimates depends on the strength and both size and structure of the sampled population (Bijma and Bastiaansen, 2014), the prediction accuracy of a low heritability trait with strong and well-estimated genetic correlations, as is the case of DBH and WD in *P. radiata* does not benefit from any additional marker selection. In contrast, the prediction accuracy of a low heritability trait with only moderate/lower and less precisely estimated genetic correlations, as in the *E. nitens* population, can benefit from the marker selection strategy proposed in this study (MVGBLUP2). Finding markers associated with the underlying genetic correlation structure can therefore potentially further improve the precision of genetic correlation estimates and thus the prediction accuracy of involved traits. However, it is worth noting that the scenarios showing the highest prediction accuracy for low heritability DBH were not supported by the model fit patterns (DIC) in either the *E. nitens* or *P. radiata* populations tested. Therefore, model fit is not a good indicator for selecting the best model in this case.

These findings indicate that the traits used in multivariate genomic analyses should, ideally, belong to the same variational module (set of traits that vary together and are independent of other traits) and show low to moderate genetic correlations in order to benefit from this approach. On the other hand, there is no further benefit of the proposed method when the estimated genetic correlation between the traits is high, such as the genetic correlation between WD and DBH in *P. radiata*. Traits in the same biological module usually show a high level of phenotypic integration, with pleiotropy likely contributing to this (Armbruster et al., 2014). Wagner et al. (2008) showed that most pleiotropic QTLs only affect a small number of traits and their effect increases with the number of traits affected. Including a large number of traits that show different genetic correlations and precision levels to their estimates can increase the efficiency of this method (PLS-CA); pleiotropic QTLs are detected through weak or negative relationships between modules which increase the precision of genetic correlation estimates and thus accuracy in the prediction of low heritability traits as shown for *E. nitens*. It is worth mentioning, however, that pleiotropic QTLs can be present even when no marker-based genetic correlations are detected between traits (Gianola et al., 2015).

The efficient implementation of genomic selection in forestry requires the consideration of at least three groups of factors: (1) the genetic architecture of measured traits, (2) the structure of the training population, and (3) the quality of the phenotypic and marker data. A trait's genetic architecture is measured through factors, such as heritability, mode of inheritance (following Fisher's infinitesimal model vs. a mixed type of inheritance with a few large effect QTLs and many small effect ones) and the effective number of chromosomal fragments (Hayes et al., 2009), which depends on the distribution of QTLs across the genome and the intensity of LD decay.

The structure of the training population [the level of shared genealogy (relatedness), co-segregation and linkage disequilibrium between markers and QTLs (Habier et al., 2013)], will determine its suitability for genomic selection. The relative contribution of each of these to success depends on the composition of the training population itself. In our study, we tested two populations with different structures. While the *E. nitens* population shows two clusters due to contributions from two seed orchards with different selection strategies (Suontama et al., 2019), *P. radiata* shows no population structure but does show family clusters (Supplementary Figure 1). Additionally, while *E. nitens* included open-pollinated progenies with recovered full-sibs and self-sibs (Klápště et al., 2017), the *P. radiata* population contained full-sib families from 24 parents (Supplementary Figure 1). Genetic connectedness is vital, and good connections among parents, families or clones are important, as is the case in any quantitative analysis (Li et al., 2018). The production and testing of large full-sib families also gives the ability to dissect additive from non-additive genetic components and examine Mendelian segregation, something which is often confounded in pedigree-based analyses (Visscher et al., 2006). The size and decay rates of linkage disequilibrium between markers and QTLs, however, plays the most important role in training when mostly unrelated or only weakly related individuals are included (Meuwissen, 2009). Since the precise estimate of genetic correlations, the most critical genetic parameter considered for this approach, requires broad genetic diversity as well as familial structure in the training population (Bijma and Bastiaansen, 2014), the optimization of structure in training populations should be carefully considered.

Additionally, both populations represent advanced generations of breeding populations which underwent several generations of selection. Such conditions might introduce decreases in the accuracy of breeding values (in terms of correlation between true breeding values and estimated breeding values), depending on selection intensity and reduction in additive genetic variance (Bijma, 2012). The reduction is more pronounced in pedigree-based analyses compared to the marker-based counterpart due to the fact that the pedigree-based scenario can predict only parental averages (which explains only a small fraction of genetic variation and true breeding values of the offspring due to selection) compared to the marker-based equivalent; predicting both parental averages and Mendelian sampling (Gorjanc et al., 2015). However, the impact of selection on accuracy of breeding values depends on the data used in the analysis. While old data from previous generations pronounces the reduction in accuracy of breeding values, new data from the current selected population minimizes the impact of selection on the accuracy of breeding values (Bijma, 2012).

### 4.2. Genomic Data Quality, Quantity, and Selection

The quality of marker data impacts directly on the ability of these markers to capture and adequately describe the genetic control and architecture of quantitative traits. The usefulness of a genomic resource is therefore a function of the number of markers, their distribution across the genome and the accuracy of the genotype calls. The platforms available for genotyping forest tree species are often driven by the nature of their genomes. In this study, the relatively small genome length of many *Eucalyptus* species (~0.56 Gb) has allowed the rapid and cost-effective development of the multi-species *Eucalyptus* SNP chip, based on SNP discovery from whole genome sequencing data (Silva-Junior et al., 2015). In contrast, the extensive size of the *Pinus radiata* genome (~25 Gb) and large amount of repetitive sequences required a different SNP discovery and genotyping approach based on reduced representation sequencing of the genome (Elshire et al., 2011; Neves et al., 2013; Telfer et al., 2018). Such approaches, or other similar techniques, such as exome capture have already been successfully implemented in other conifer species (Gamal El-Dien et al., 2015; Ratcliffe et al., 2015; Bartholomé et al., 2016; Isik et al., 2016; Lenz et al., 2017; Chen et al., 2018).

The large amount of genomic data obtained in genomic selection studies can contain some level of redundancy, which can negatively affect the accuracy of breeding values (Habier et al., 2013) and might necessitate variable selection approaches. Ballesta et al. (2018) found an advantage to dimensionality reduction and variable selection, improving prediction accuracy of low-to-moderate heritability traits in a single-trait evaluation in a *Eucalyptus globulus* population. Our strategy resulted in the highest prediction accuracy for the primary trait when ~ 66% (considering only positive loadings) and ~ 35% (considering both positive and negative loadings) of markers were included in the marker-based relationship matrix in *E. nitens*, and ~94% and 99% markers in *P. radiata* (Table 4, Supplementary Tables 1, 4). Lippert et al. (2013) found that the pre-selection of QTL-related markers, or at least increasing the proportion of such markers over uninformative ones was an advantage and increased the accuracy of predicted genomic breeding values. Several other approaches have been examined, using marker weights developed using either Bayesian inference (Kemper et al., 2018) or results from previous QTL mapping or association studies (Fragomeni et al., 2017). The proportion of markers selected reflects the genetic complexity of the trait under study. For example, Müller et al. (2017) found that 5,000–10,000 markers (representing ~40–60% of full marker data) were sufficient to capture the major proportion of trait heritability and reach the same prediction accuracy compared to using the all marker dataset. Similarly, Resende et al. (2012) found an advantage to using reduced numbers of markers in traits, such as wood specific gravity (~5% of total marker data) and resistance to Fusiform rust [gall volume (~2% of total marker data) and presence or absence of rust (~7% of the total marker data)]. Additionally, Chen et al. (2018) found that the structure of the training population (full-sib vs. half-sib families) defines the number of selected markers needed to reach prediction accuracies equivalent to using full marker data. While for a full-sib structure 4,000–8,000 markers was found to be sufficient, a half-sib structure required all 100,000 markers to reach the maximum achievable prediction accuracy. However, the selection of informative markers was performed using only single trait approaches and different standards of genomic resources.

Similar to our approach, several proposed strategies have been developed within a Bayesian multivariate framework (Cheng et al., 2018; Karaman et al., 2018). Karaman et al. (2018) found there was benefit to assigning specific weight to blocks of fixed numbers of markers rather than to each marker individually. Our approach allows for the selection of markers associated with genomic regions related to shared underlying genetic components across investigated traits, without any prior definition of the block length. Since our approach associates the markers with underlying structure rather than with each trait involved in the study, it shows benefits even in the case of sparse marker arrays as used in this study. However, the presence of full phenotypic data is required to perform marker selection through PLS-CA, and thus the investigated traits have to be screened at an operational scale.

The strategy proposed in this study does not attempt to improve the accuracy of all traits involved in the analysis but only those with low heritabilities, taking advantage of the genetic covariances common across all investigated traits. The latent variables created through PLS-CA analysis (Tenenhaus, 1998; Sanchez and Sanchez, 2012) tend to extract the common part of variances in both the trait and marker data by maximization of covariance between latent variables. Ideally, the algorithm searches for bridges between variational modules (group of traits that vary together) and functional modules (group of genes/proteins that are coordinated to perform semi-autonomous functions) (Kliebenstein, 2011). However, the efficiency of finding such bridges depends on adequate representation of the genome through marker data. The investigation of marker loadings associated with the latent variables can identify those markers important for explaining the variance captured by each latent variable. Additionally, this investigation will also indirectly identify the markers which most likely explain variance explaining the behavior of the corresponding latent variable derived from phenotypic data. As mentioned above, the efficiency of the proposed strategy depends on the level of integration and modularity between the traits under study. Therefore, the selection of traits included in the analysis should take into consideration their biological connection and their heritabilities.

In general, the magnitude of genetic correlations between traits has an impact on the accuracy of breeding values (Jia and Jannink, 2012). However, the method proposed in this study benefited from improvement in the precision of genetic correlation structure through marker selection only when pairwise correlations were low or moderate. In contrast, no additional benefit beyond the commonly used model (MVGBLUP) was found in a population with well-estimated strong genetic correlation between primary (DBH) and other (WD) traits. Pleiotropic QTLs, however, can be included in the underlying genetic structures used in the analysis, even where no genetic correlations between traits are detected using genetic markers. Therefore, marker-based genetic correlations can be misleading to provide inference about their causes when knowledge about LD between markers and QTLs is poor or non-existing (Gianola et al., 2015).

## 5. Conclusions

The approach proposed in this study selects markers aligned to the underlying dimensions extracted from a trait's covariance structure rather than investigating associations between markers with each trait, which allows for improvements even with sparse marker arrays. This method is suitable for improving the accuracy of low heritability traits where genetic correlations between traits are low/moderate in magnitude and low accuracy. In contrast, when the population shows a strong genetic correlation between the primary trait (DBH in this study) and other moderately heritable traits, this approach does not show benefit beyond that observed with the multivariate model using all genetic markers. One drawback is that this approach requires all individuals in the training population to be phenotyped for all traits included in the analysis to perform the marker selection procedure (PLS-CA).

## Data Availability Statement

The datasets generated for this study can be found in the ZENODO data repository: doi: 10.5281/zenodo.4040042.

## Author Contributions

JK performed the analyses and drafted the manuscript. MS, HD, ET, NG, and YL designed the study, assisted with drafting the manuscript, and secured the funding. RM developed the phenotyping protocols for wood quality attributes. All authors significantly contributed to the current study.

## Conflict of Interest

The authors declare that this study received funding from NZ Ministry of Business, Innovation and Employment and Radiata Pine Breeding Company Ltd. The Radiata Pine Breeding Company Ltd. was involved in phenotypic data collection. The funders were not involved in the study design, analysis, interpretation of data, the writing of this article or the decision to submit it for publication. JK was employed by the company Scion. The remaining authors declare that the research was conducted in the absence of any commercial or financial relationships that could be construed as a potential conflict of interest.
