# Supplementary material for: Marker Selection in Multivariate Genomic Prediction Improves Accuracy of Low Heritability Traits
Source: Front Genet. 2020 Oct 30;11:499094. doi: 10.3389/fgene.2020.499094 (PMC7662070; doi:10.3389/fgene.2020.499094)
Supplement: Supplementary file 1 [file Data_Sheet_1.PDF]

Supplementary Material

1 SUPPLEMENTARY TABLES AND FIGURES

	Species	*Eucalyptus*					*Pinus*				
Scen	Prop	P10	P20	P30	P40	P50	P10	P20	P30	P40	P50
Pos	C1	18444.6	18350.8	18306.5	18256.2	18217.1	9435.1	7572.6	7495.3	7349.7	7459.3
	C2	18372.3	18296.9	18246.0	18194.3	18158.5	7725.0	7487.5	7388.3	7416.4	7371.8
	C3	18283.8	18228.7	18195.6	18160.1	18128.0	7569.6	7457.1	7425.2	7330.3	7308.5
	C4	18263.8	18206.6	18176.2	18147.4	18121.3	7575.2	7466.6	7409.9	7394.2	7385.4
	C5	18243.3	18186.3	18163.4	18139.6	18121.1	7522.6	7445.8	7390.9	7339.7	7337.7
	C6	18213.3	18167.6	18146.1	18125.7	18111.4	NA	NA	NA	NA	NA
	C7	18202.5	18165.8	18141.0	18116.7	18107.2	NA	NA	NA	NA	NA
Pos + Neg	C1	18353.9	18258.8	18209.2	18148.4	18104.9	7665.3	7364.3	7349.3	7357.4	7315.1
	C2	18292.5	18212.3	18159.4	18120.7	18104.8	7639.1	7435.0	7378.6	7349.3	7343.9
	C3	18225.5	18157.7	18128.0	18109.1	18104.1	7529.1	7337.4	7321.8	7322.9	7320.2
	C4	18204.7	18140.9	18112.9	18104.6	18103.0	7476.6	7414.3	7299.5	7350.9	7283.2
	C5	18186.9	18130.2	18106.8	18104.5	18104.0	7384.6	7379.3	7371.7	7340.6	7350.7
	C6	18161.6	18124.4	18106.6	18104.8	18104.3	NA	NA	NA	NA	NA
	C7	18144.6	18120.7	18104.8	18103.7	18103.8	NA	NA	NA	NA	NA

Table S1. Average deviance information criterion (DIC - lower is better) across 10 folds obtained at each combination of number of latent variables (C1, C2, C3, ..) and proportion of markers (10th percentile - P10, 20th percentile - P20, ..) selected to construct marker-based relationship matrix: using markers having only positive loadings (upper part) or both positive and negative loadings (bottom part).

Scenario	Positive loadings only						
	**TS**	**WD**	**DBH**	**ST1**	**ST2**	**GS1**	**GS2**
C1P10	0.587 (0.077)	0.719 (0.061)	-0.266 (0.562)	0.440 (0.094)	0.411 (0.098)	0.530 (0.106)	0.596 (0.095)
C1P20	0.602 (0.079)	0.742 (0.064)	-0.051 (0.590)	0.468 (0.096)	0.432 (0.089)	0.561 (0.066)	0.617 (0.051)
C1P30	0.625 (0.067)	0.754 (0.058)	0.298 (0.507)	0.482 (0.088)	0.440 (0.093)	0.569 (0.065)	0.622 (0.057)
C1P40	0.634 (0.065)	0.760 (0.050)	0.393 (0.244)	0.490 (0.095)	0.422 (0.100)	0.594 (0.078)	0.647 (0.058)
C1P50	0.644 (0.058)	0.755 (0.055)	0.349 (0.365)	0.509 (0.093)	0.432 (0.105)	0.616 (0.068)	**0.666** (0.050)
C2P10	0.609 (0.064)	0.750 (0.047)	0.437 (0.381)	0.475 (0.066)	0.396 (0.100)	0.574 (0.103)	0.635 (0.085)
C2P20	0.624 (0.062)	**0.762** (0.049)	0.294 (0.535)	0.517 (0.085)	0.431 (0.095)	0.584 (0.077)	0.638 (0.062)
C2P30	0.637 (0.053)	0.754 (0.054)	0.274 (0.491)	0.518 (0.085)	**0.446** (0.118)	0.597 (0.077)	0.642 (0.068)
C2P40	0.645 (0.056)	0.753 (0.056)	0.233 (0.402)	0.522 (0.085)	0.434 (0.122)	0.609 (0.078)	0.651 (0.078)
C2P50	0.650 (0.058)	0.750 (0.059)	0.137 (0.510)	0.524 (0.083)	0.434 (0.126)	0.612 (0.074)	0.650 (0.074)
C3P10	0.637 (0.052)	0.753 (0.055)	0.435 (0.521)	0.501 (0.074)	0.410 (0.123)	0.601 (0.083)	0.644 (0.075)
C3P20	0.647 (0.049)	0.762 (0.051)	0.515 (0.309)	0.520 (0.080)	0.426 (0.121)	0.601 (0.072)	0.651 (0.061)
C3P30	0.650 (0.047)	0.759 (0.053)	**0.576** (0.241)	0.525 (0.074)	0.434 (0.137)	0.604 (0.072)	0.650 (0.065)
C3P40	0.653 (0.051)	0.759 (0.053)	0.571 (0.257)	0.532 (0.081)	0.431 (0.136)	0.609 (0.068)	0.657 (0.064)
C3P50	0.653 (0.056)	0.758 (0.050)	0.444 (0.293)	0.537 (0.079)	0.432 (0.132)	0.613 (0.068)	0.659 (0.066)
C4P10	0.637 (0.043)	0.754 (0.049)	0.395 (0.497)	0.507 (0.087)	0.413 (0.132)	0.610 (0.092)	0.647 (0.083)
C4P20	0.646 (0.049)	0.760 (0.047)	0.378 (0.400)	0.521 (0.088)	0.425 (0.128)	0.605 (0.079)	0.648 (0.070)
C4P30	0.649 (0.049)	0.759 (0.050)	0.454 (0.416)	0.532 (0.088)	0.441 (0.137)	0.610 (0.069)	0.654 (0.062)
C4P40	0.652 (0.053)	0.758 (0.046)	0.394 (0.383)	0.535 (0.088)	0.439 (0.131)	0.615 (0.071)	0.658 (0.061)
C4P50	0.652 (0.059)	0.758 (0.045)	0.420 (0.301)	0.538 (0.081)	0.436 (0.135)	0.613 (0.071)	0.660 (0.070)
C5P10	0.643 (0.043)	0.760 (0.054)	0.271 (0.374)	0.510 (0.080)	0.413 (0.128)	0.606 (0.087)	0.646 (0.085)
C5P20	0.644 (0.054)	0.760 (0.045)	0.161 (0.352)	0.530 (0.080)	0.433 (0.130)	0.611 (0.079)	0.650 (0.074)
C5P30	0.651 (0.055)	0.758 (0.050)	0.254 (0.377)	0.537 (0.082)	0.440 (0.135)	0.613 (0.070)	0.655 (0.064)
C5P40	0.653 (0.053)	0.758 (0.046)	0.368 (0.446)	0.536 (0.081)	0.438 (0.133)	0.616 (0.069)	0.658 (0.059)
C5P50	0.655 (0.059)	0.761 (0.044)	0.445 (0.320)	0.538 (0.081)	0.436 (0.132)	0.614 (0.074)	0.662 (0.061)
C6P10	0.645 (0.043)	0.765 (0.054)	0.231 (0.376)	0.512 (0.085)	0.406 (0.124)	0.611 (0.086)	0.648 (0.087)
C6P20	0.645 (0.053)	0.761 (0.044)	0.407 (0.278)	0.532 (0.082)	0.429 (0.129)	0.613 (0.080)	0.653 (0.073)
C6P30	0.653 (0.056)	0.761 (0.045)	0.465 (0.255)	0.539 (0.082)	0.436 (0.132)	0.613 (0.073)	0.656 (0.063)
C6P40	0.655 (0.057)	0.760 (0.043)	0.387 (0.334)	0.539 (0.084)	0.435 (0.133)	0.617 (0.071)	0.663 (0.063)
C6P50	0.655 (0.060)	0.762 (0.043)	0.409 (0.295)	0.541 (0.082)	0.436 (0.133)	0.618 (0.072)	0.664 (0.064)
C7P10	0.648 (0.044)	0.761 (0.062)	0.310 (0.303)	0.515 (0.092)	0.412 (0.125)	0.606 (0.090)	0.643 (0.084)
C7P20	0.650 (0.054)	0.759 (0.045)	0.341 (0.336)	0.534 (0.081)	0.432 (0.130)	0.616 (0.080)	0.656 (0.072)
C7P30	**0.657** (0.057)	0.760 (0.044)	0.380 (0.322)	0.541 (0.080)	0.438 (0.131)	0.615 (0.071)	0.658 (0.062)
C7P40	0.657 (0.059)	0.761 (0.043)	0.335 (0.311)	0.542 (0.079)	0.437 (0.131)	0.617 (0.073)	0.661 (0.067)
C7P50	0.657 (0.059)	0.762 (0.043)	0.526 (0.234)	**0.544** (0.081)	0.436 (0.131)	**0.619** (0.074)	0.664 (0.065)

Table S2. Prediction ability and its standard deviation (in parenthesis) for each trait and SNP selection scenario using marker having only positive loadings in *E.nitens*.

Scenario	Positive + negative loadings						
	**TS**	**WD**	**DBH**	**ST1**	**ST2**	**GS1**	**GS2**
C1P10	0.625 (0.063)	0.746 (0.038)	-0.067 (0.502)	0.495 (0.113)	0.423 (0.114)	0.579 (0.102)	0.636 (0.087)
C1P20	0.637 (0.070)	0.759 (0.043)	0.330 (0.371)	0.513 (0.101)	**0.447** (0.115)	0.600 (0.073)	0.651 (0.044)
C1P30	0.647 (0.066)	0.764 (0.042)	0.415 (0.305)	0.520 (0.084)	0.445 (0.130)	0.599 (0.070)	0.647 (0.053)
C1P40	0.653 (0.061)	0.769 (0.039)	0.376 (0.342)	0.532 (0.082)	0.430 (0.133)	0.598 (0.073)	0.648 (0.059)
C1P50	0.659 (0.059)	0.763 (0.042)	0.478 (0.250)	0.543 (0.080)	0.436 (0.132)	0.618 (0.072)	0.664 (0.063)
C2P10	0.642 (0.059)	0.766 (0.035)	**0.595** (0.353)	0.523 (0.078)	0.414 (0.107)	0.604 (0.085)	0.660 (0.073)
C2P20	0.650 (0.056)	**0.771** (0.036)	0.515 (0.457)	0.534 (0.089)	0.431 (0.124)	0.614 (0.069)	0.664 (0.056)
C2P30	0.651 (0.056)	0.768 (0.039)	0.433 (0.358)	0.536 (0.084)	0.443 (0.135)	0.610 (0.074)	0.657 (0.066)
C2P40	0.657 (0.056)	0.764 (0.041)	0.547 (0.221)	0.539 (0.082)	0.434 (0.135)	0.613 (0.078)	0.662 (0.068)
C2P50	0.659 (0.059)	0.763 (0.043)	0.459 (0.278)	0.544 (0.081)	0.435 (0.133)	0.618 (0.072)	0.664 (0.067)
C3P10	0.652 (0.059)	0.761 (0.042)	0.502 (0.364)	0.534 (0.079)	0.416 (0.128)	**0.621** (0.070)	0.666 (0.061)
C3P20	0.655 (0.054)	0.766 (0.042)	0.459 (0.296)	0.536 (0.082)	0.426 (0.134)	0.617 (0.070)	0.663 (0.061)
C3P30	0.659 (0.056)	0.764 (0.043)	0.469 (0.321)	0.543 (0.077)	0.436 (0.136)	0.616 (0.075)	0.661 (0.067)
C3P40	0.659 (0.059)	0.763 (0.043)	0.452 (0.280)	0.544 (0.080)	0.434 (0.134)	0.618 (0.071)	0.663 (0.065)
C3P50	0.658 (0.059)	0.762 (0.043)	0.400 (0.224)	0.545 (0.080)	0.436 (0.133)	0.616 (0.074)	0.660 (0.066)
C4P10	0.646 (0.061)	0.762 (0.040)	0.494 (0.246)	0.537 (0.081)	0.421 (0.138)	0.617 (0.082)	0.664 (0.072)
C4P20	0.656 (0.057)	0.767 (0.044)	0.402 (0.273)	0.538 (0.082)	0.433 (0.138)	0.618 (0.076)	**0.665** (0.063)
C4P30	**0.660** (0.057)	0.763 (0.043)	0.475 (0.285)	0.544 (0.082)	0.437 (0.135)	0.618 (0.073)	0.665 (0.067)
C4P40	0.659 (0.058)	0.763 (0.042)	0.445 (0.268)	0.543 (0.082)	0.434 (0.133)	0.617 (0.072)	0.662 (0.066)
C4P50	0.658 (0.059)	0.762 (0.041)	0.387 (0.327)	0.543 (0.080)	0.436 (0.134)	0.618 (0.072)	0.663 (0.068)
C5P10	0.650 (0.062)	0.764 (0.041)	0.338 (0.251)	0.538 (0.079)	0.423 (0.136)	0.612 (0.075)	0.656 (0.068)
C5P20	0.656 (0.058)	0.767 (0.042)	0.419 (0.213)	0.542 (0.079)	0.438 (0.132)	0.619 (0.068)	0.663 (0.061)
C5P30	0.660 (0.057)	0.763 (0.043)	0.385 (0.251)	0.545 (0.081)	0.435 (0.132)	0.614 (0.071)	0.660 (0.066)
C5P40	0.659 (0.057)	0.762 (0.043)	0.428 (0.321)	0.544 (0.080)	0.436 (0.133)	0.620 (0.070)	0.664 (0.064)
C5P50	0.659 (0.059)	0.762 (0.043)	0.451 (0.372)	0.544 (0.081)	0.435 (0.133)	0.618 (0.071)	0.664 (0.064)
C6P10	0.652 (0.062)	0.768 (0.041)	0.424 (0.302)	0.544 (0.080)	0.420 (0.135)	0.618 (0.075)	0.663 (0.071)
C6P20	0.653 (0.062)	0.764 (0.043)	0.466 (0.317)	0.542 (0.078)	0.436 (0.132)	0.618 (0.073)	0.664 (0.065)
C6P30	0.658 (0.058)	0.762 (0.042)	0.472 (0.313)	0.545 (0.081)	0.436 (0.133)	0.617 (0.074)	0.662 (0.066)
C6P40	0.660 (0.059)	0.762 (0.043)	0.428 (0.363)	0.544 (0.080)	0.435 (0.132)	0.618 (0.072)	0.663 (0.062)
C6P50	0.658 (0.060)	0.762 (0.044)	0.397 (0.392)	0.543 (0.080)	0.434 (0.133)	0.618 (0.073)	0.663 (0.062)
C7P10	0.655 (0.059)	0.765 (0.043)	0.411 (0.264)	**0.547** (0.085)	0.424 (0.130)	0.613 (0.076)	0.656 (0.073)
C7P20	0.655 (0.060)	0.762 (0.043)	0.398 (0.371)	0.543 (0.080)	0.434 (0.131)	0.618 (0.068)	0.665 (0.059)
C7P30	0.658 (0.059)	0.763 (0.042)	0.442 (0.282)	0.544 (0.081)	0.435 (0.133)	0.618 (0.071)	0.662 (0.063)
C7P40	0.658 (0.059)	0.762 (0.043)	0.436 (0.310)	0.544 (0.081)	0.436 (0.133)	0.617 (0.072)	0.663 (0.064)
C7P50	0.659 (0.059)	0.762 (0.043)	0.422 (0.339)	0.543 (0.080)	0.436 (0.133)	0.617 (0.068)	0.664 (0.061)

Table S3. Prediction ability and its standard deviation (in parenthesis) for each trait and SNP selection scenario using markers having both positive and negative loadings in *E.nitens*.

	Positive loadings only				
Scenario	**BR9**	**DBH**	**ST9**	**WD**	**PME**
C1P10	0.570 (0.130)	0.537 (0.095)	0.429 (0.112)	0.593 (0.070)	0.499 (0.088)
C1P20	0.564 (0.141)	0.562 (0.098)	0.425 (0.121)	**0.627** (0.064)	0.535 (0.106)
C1P30	0.549 (0.150)	0.552 (0.107)	0.431 (0.126)	0.610 (0.055)	0.518 (0.111)
C1P40	0.558 (0.139)	0.587 (0.074)	0.434 (0.132)	0.611 (0.061)	0.524 (0.110)
C1P50	0.563 (0.141)	0.585 (0.086)	0.429 (0.155)	0.618 (0.052)	0.529 (0.124)
C2P10	0.581 (0.116)	0.565 (0.065)	0.435 (0.128)	0.599 (0.061)	0.522 (0.103)
C2P20	0.576 (0.123)	0.601 (0.063)	0.432 (0.134)	0.624 (0.060)	0.537 (0.117)
C2P30	0.567 (0.127)	0.601 (0.067)	0.438 (0.137)	0.623 (0.053)	0.538 (0.119)
C2P40	0.570 (0.133)	0.611 (0.058)	0.436 (0.139)	0.622 (0.056)	0.532 (0.121)
C2P50	0.572 (0.129)	**0.616** (0.058)	0.435 (0.146)	0.626 (0.056)	0.530 (0.120)
C3P10	0.581 (0.129)	0.584 (0.062)	0.437 (0.141)	0.611 (0.055)	0.532 (0.109)
C3P20	0.579 (0.131)	0.605 (0.056)	0.435 (0.137)	0.618 (0.052)	0.531 (0.113)
C3P30	0.571 (0.136)	0.604 (0.074)	0.437 (0.141)	0.616 (0.053)	0.528 (0.113)
C3P40	0.569 (0.136)	0.615 (0.056)	0.434 (0.139)	0.621 (0.055)	0.523 (0.115)
C3P50	0.569 (0.134)	0.611 (0.053)	0.436 (0.138)	0.620 (0.048)	0.534 (0.120)
C4P10	**0.586** (0.134)	0.593 (0.052)	0.442 (0.144)	0.606 (0.051)	**0.542** (0.113)
C4P20	0.573 (0.138)	0.607 (0.061)	0.434 (0.143)	0.618 (0.056)	0.531 (0.118)
C4P30	0.569 (0.137)	0.611 (0.058)	0.430 (0.146)	0.619 (0.055)	0.528 (0.115)
C4P40	0.566 (0.137)	0.609 (0.049)	0.433 (0.143)	0.617 (0.050)	0.526 (0.119)
C4P50	0.566 (0.138)	0.605 (0.062)	0.433 (0.140)	0.615 (0.064)	0.529 (0.124)
C5P10	0.584 (0.133)	0.562 (0.100)	**0.446** (0.149)	0.601 (0.057)	0.528 (0.115)
C5P20	0.572 (0.141)	0.607 (0.055)	0.435 (0.144)	0.616 (0.053)	0.533 (0.113)
C5P30	0.564 (0.139)	0.608 (0.059)	0.430 (0.148)	0.616 (0.057)	0.526 (0.111)
C5P40	0.565 (0.139)	0.613 (0.069)	0.434 (0.142)	0.619 (0.070)	0.525 (0.124)
C5P50	0.566 (0.136)	0.620 (0.058)	0.434 (0.143)	0.626 (0.055)	0.536 (0.111)

Table S4. Prediction ability and its standard deviation (in parenthesis) for each trait and SNP selection scenario using markers having only positive loadings in *P.radiata*.

Scenario	Positive + negative loadings				
	**BR9**	**DBH**	**ST9**	**WD**	**PME**
C1P10	0.571 (0.143)	0.594 (0.075)	**0.436** (0.119)	0.595 (0.071)	0.514 (0.105)
C1P20	0.577 (0.151)	**0.626** (0.048)	0.417 (0.129)	**0.631** (0.044)	**0.543** (0.108)
C1P30	0.565 (0.149)	0.615 (0.055)	0.422 (0.127)	0.623 (0.053)	0.521 (0.114)
C1P40	0.561 (0.148)	0.607 (0.056)	0.430 (0.129)	0.614 (0.056)	0.525 (0.113)
C1P50	0.569 (0.135)	0.609 (0.056)	0.430 (0.142)	0.619 (0.054)	0.527 (0.120)
C2P10	**0.589** (0.123)	0.606 (0.062)	0.429 (0.134)	0.613 (0.058)	0.530 (0.109)
C2P20	0.578 (0.139)	0.606 (0.054)	0.422 (0.141)	0.617 (0.051)	0.526 (0.110)
C2P30	0.569 (0.138)	0.608 (0.061)	0.431 (0.137)	0.619 (0.060)	0.526 (0.117)
C2P40	0.569 (0.140)	0.625 (0.059)	0.430 (0.143)	0.631 (0.057)	0.534 (0.122)
C2P50	0.570 (0.135)	0.621 (0.056)	0.432 (0.143)	0.624 (0.056)	0.534 (0.120)
C3P10	0.579 (0.139)	0.586 (0.096)	0.432 (0.139)	0.628 (0.052)	0.532 (0.123)
C3P20	0.572 (0.139)	0.610 (0.068)	0.428 (0.140)	0.625 (0.049)	0.530 (0.120)
C3P30	0.573 (0.131)	0.605 (0.054)	0.426 (0.142)	0.623 (0.050)	0.529 (0.113)
C3P40	0.568 (0.136)	0.618 (0.060)	0.431 (0.145)	0.626 (0.059)	0.531 (0.118)
C3P50	0.570 (0.135)	0.609 (0.062)	0.431 (0.143)	0.616 (0.061)	0.529 (0.118)
C4P10	0.582 (0.135)	0.604 (0.060)	0.431 (0.135)	0.612 (0.057)	0.533 (0.123)
C4P20	0.572 (0.140)	0.617 (0.055)	0.430 (0.137)	0.628 (0.055)	0.535 (0.116)
C4P30	0.568 (0.137)	0.615 (0.060)	0.432 (0.142)	0.623 (0.060)	0.528 (0.120)
C4P40	0.568 (0.137)	0.605 (0.058)	0.432 (0.142)	0.613 (0.056)	0.523 (0.120)
C4P50	0.568 (0.137)	0.617 (0.062)	0.433 (0.140)	0.626 (0.062)	0.526 (0.113)
C5P10	0.580 (0.136)	0.611 (0.062)	0.434 (0.138)	0.629 (0.056)	0.537 (0.114)
C5P20	0.571 (0.138)	0.623 (0.054)	0.431 (0.140)	0.627 (0.058)	0.539 (0.127)
C5P30	0.568 (0.136)	0.616 (0.066)	0.429 (0.144)	0.622 (0.062)	0.531 (0.118)
C5P40	0.567 (0.138)	0.611 (0.057)	0.433 (0.143)	0.620 (0.057)	0.529 (0.117)
C5P50	0.567 (0.137)	0.610 (0.063)	0.431 (0.143)	0.617 (0.063)	0.523 (0.117)

Table S5. Prediction ability and its standard deviation (in parenthesis) for each trait and SNP selection scenario using markers having both positive and negative loadings in *P.radiata*.

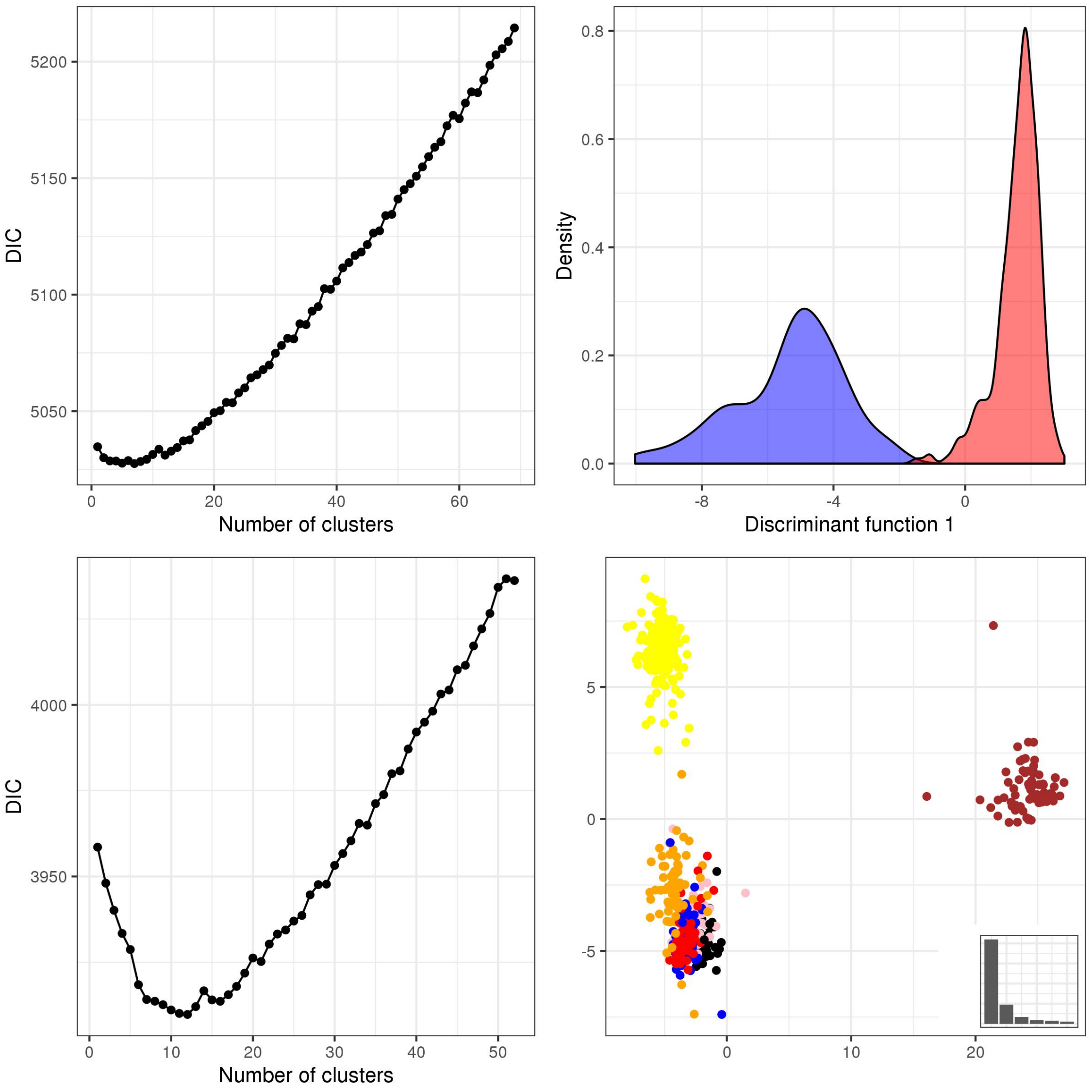

Figure S1. Determination of population structure through discriminant analysis of principal components (DAPC); DIC criterion detecting the best scenario for the number of clusters (left plots) and distribution of clusters (right plots) in *E.nitens* (upper plots) and *P.radiata* (bottom plots)

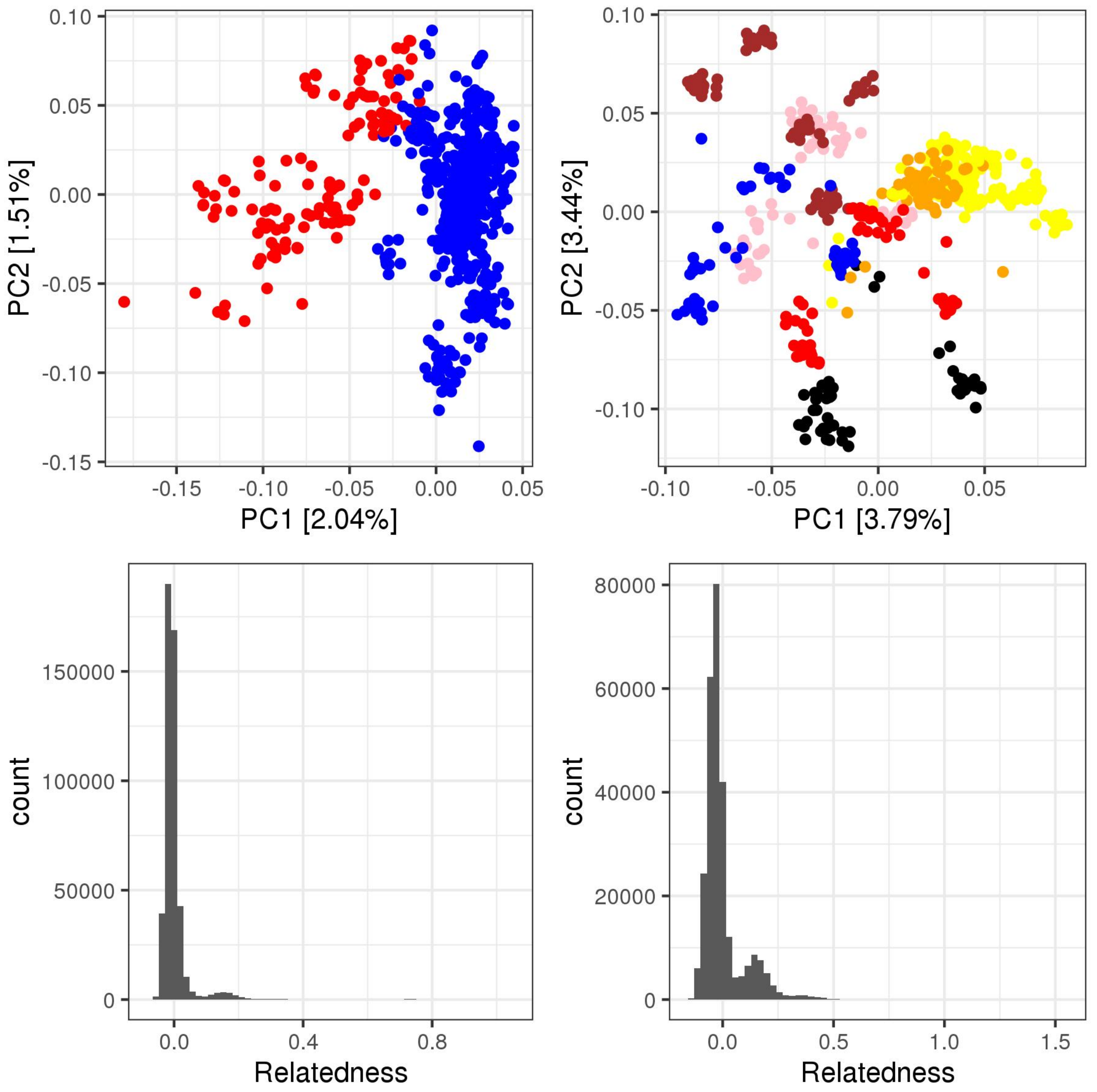

Figure S2. Population structure of *E.nitens* (upper left plot) and *P.radiata* (upper right plot) and histogram of relatedness in *E. nitens* (bottom left plot) and *P. radiata* (bottom right plot)

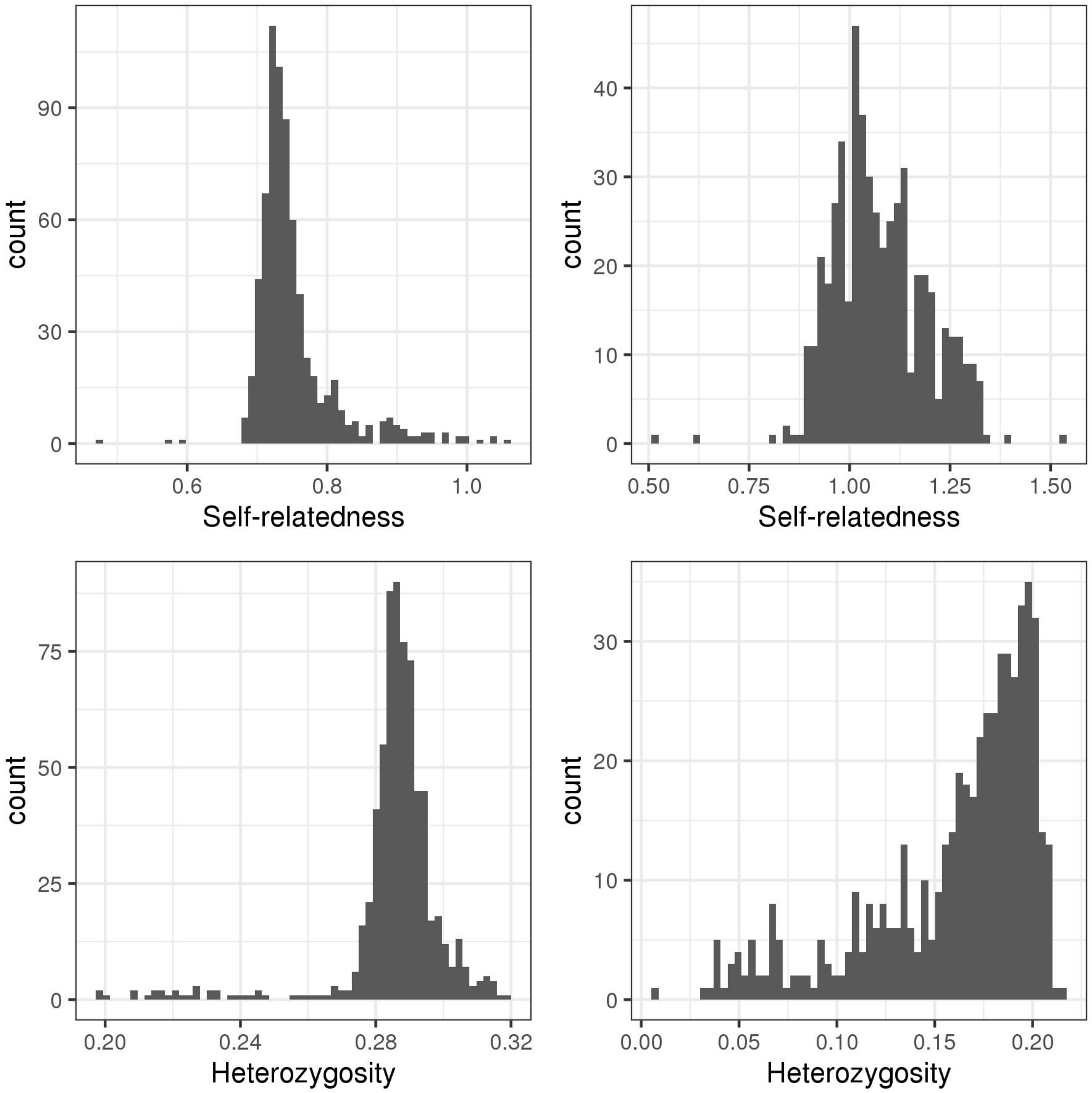

Figure S3. Self-relatedness of *E.nitens* (upper left plot) and *P.radiata* (upper right plot) population and histogram of sample heterozygosity in *E. nitens* (bottom left plot) and *P. radiata* (bottom right plot) population
